# Eighteenth Annual Session

**Published:** 1867-06

**Authors:** 


### AMERICAN MEDICAL ASSOCIATION—EIGHTEENTH ANNUAL SESSION.

This Association met Tuesday, May 7th, at 11 o'clock, A.M., in Hopkin's Hall. For an hour previous the reception room adjoining had been crowded with delegates, presenting their credentials to the Secretary, Dr. Wm. B. Atkinson, of Philadelphia, enrolling their names, and receiving from the Cincinnati Committees such attentions as the occasion has called forth.

The Committee of Arrangements, Dr. John A. Murphy, Chairman, Drs. W. W. Dawson, J. P. Walker, R. R. McIlvaine, W. T. Brown, J. S. Unzicker, and James Graham, presented each delegate or visitor, with a neatly-printed pocket pamphlet with a card-board cover, on the front page of which is printed "American Medical Association, Annual Meeting, Cincinnati, May 7th, 1867. This card will admit Dr. —— —— to the within named places." On the second page the following: "Places of Public Interest—Court House, City Jail, Young Men's Mercantile Library, Chamber of Commerce, Theological Library, School Library, Mechanics' Institute, Longworth's Wine Cellar, House of Refuge, Protestant Orphan Asylum, Catholic Orphan Asylum, Spring Grove Cemetery, Longview Lunatic Asylum, City Buildings, Greenwood's Foundry, Water Works, Medical College of Ohio, Miami Medical College, Good Samaritan Hospital, Commercial Hospital, Steam Fire Engines."

Next is a copy of Mr. E. Mendenhall's map of the city, stitched in and folded up in good style. Then follow sixteen pages of matter descriptive of the "Places of Public Interest" before mentioned, and numerous others, making a very creditable City Guide, for the use of strangers visiting our town.

The Committee on Entertainment, Dr. David Judkins, Chairman, Drs. C. G. Comegys, W. H. Taylor, C. P. Wilson, T. Carroll, J. S. Dodge, and W. E. Foote, presented each delegate with a ticket to the entertainment, or banquet, at Melodeon Hall, last night, and another to the excursion on the magnificent Louisville mail steamer America on Thursday.

### PROCEEDINGS OF THE ASSOCIATION.

At eleven o'clock the President of the Association, Dr.

Henry F. Askew, of Delaware, called the meeting to order. Rev. Dr. Storrs, of this city, offered a prayer.

Dr. John A. Murphy, as Chairman of the Committee of Arrangements, then made the following

